# Expanding networks of RNA virus evolution

**DOI:** 10.1186/1741-7007-10-54

**Published:** 2012-06-20

**Authors:** Eugene V Koonin, Valerian V Dolja

**Affiliations:** 1 National Center for Biotechnology Information, National Institutes of Health, Bethesda, MD 20894, USA; 2 Department of Botany and Plant Pathology and Center for Genome Research and Biocomputing, Oregon State University, Corvallis, OR 97331, USA

## Abstract

In a recent *BMC Evolutionary Biology *article, Huiquan Liu and colleagues report two new genomes of double-stranded RNA (dsRNA) viruses from fungi and use these as a springboard to perform an extensive phylogenomic analysis of dsRNA viruses. The results support the old scenario of polyphyletic origin of dsRNA viruses from different groups of positive-strand RNA viruses and additionally reveal extensive horizontal gene transfer between diverse viruses consistent with the network-like rather than tree-like mode of viral evolution. Together with the unexpected discoveries of the first putative archaeal RNA virus and a RNA-DNA virus hybrid, this work shows that RNA viral genomics has major surprises to deliver.

See research article: http://www.biomedcentral.com/1471-2148/12/91

## Commentary

Viruses are the most abundant and genetically diverse biological entities on earth as demonstrated by recent metagenomic studies [1]. Unlike cellular life forms that all possess the same basic mechanism of genome replication and expression based upon double-stranded (ds)DNA genome and positive-strand messenger (plus) RNA, viruses use all forms of genetic material (positive-strand, negative-strand and dsRNA, single-stranded (ss)DNA, dsDNA) and execute virtually all conceivable genomic strategies (RNA or DNA replication and transcription, as well as reverse transcription of RNA to DNA). In particular, viruses with RNA genomes that do not go through a DNA stage in their reproduction are the simplest genetic elements, reminiscent of the putative primordial RNA world.

Owing to their (relatively) small genomes and often rapid reproduction, diverse viral genomes were characterized in the early days of genomics, and many unexpected evolutionary relationships have been revealed between viruses with different genomic strategies that infect widely different hosts. A small set of virus hallmark genes encoding proteins essential for virus replication and morphogenesis form different combinations in diverse viruses but are absent from cellular genomes [2]. Along with lineage-specific genes present in subsets of viruses, the hallmark genes account for a rich network of evolutionary connections against the background of the extreme diversity of viruses. In the last few years, new technologies, in particular the rapid progress of metagenomics (indiscriminate sequencing of environmental DNA samples), have revealed many surprising novelties in the virus world. Perhaps the prime example is the discovery of giant viruses and their parasites, the virophages [3]. However, unexpected findings with substantial implications are also being reported for the much smaller and simpler RNA viruses.

In a recent *BMC Evolutionary Biology *article, Huiquan Liu and colleagues [4] report the genomes of two novel dsRNA viruses from fungi and proceed to place these viruses in the overall context of dsRNA virus evolution. dsRNA viruses have been isolated from animals, plants, protists and bacteria but are particularly characteristic in fungi where they constitute the majority of the known viruses. Some of the dsRNA viruses form typical virions with genomes encased in a capsid whereas others are capsid-less, plasmid-like genetic elements. On the whole, there is little doubt that dsRNA viruses make up a polyphyletic assemblage, with different members having evolved from different lineages of positive-strand RNA viruses on multiple, independent occasions [5]. For instance, three families of capsid-less dsRNA viruses that reproduce in fungi, plants or fungal mitochondria clearly originate from three distinct groups of positive-strand RNA viruses: *Hypoviridae *infecting plant pathogenic fungi from plant potyviruses [6]; *Endornaviridae *from alphavirus-like superfamily positive-strand RNA viruses of plants [7]; and fungal mitochondrial narnaviruses from bacterial positive-strand RNA viruses (*Leviviridae*) [8]. The origins of the rest of the dsRNA viruses are murkier, although the families *Totiviridae *and *Partitiviridae *have been tentatively linked to different groups of picornavirus-like positive-strand RNA viruses [6].

The only gene that is conserved in all dsRNA viruses along with positive-strand RNA viruses is the RNA-dependent RNA polymerase (RdRp), and most of the conclusions on evolution of RNA viruses are based on RdRp phylogeny supplemented by phylogenies of other genes that are conserved in subsets of viruses and comparative analysis of genome organization [6]. Huiquan Liu and colleagues [4] present the most complete phylogeny so far of the RdRp for dsRNA viruses and overlay the tree with the data on genome organization, virion structure and host range of the respective viruses. The result is fascinating: several clades of RdRps encompass viruses infecting diverse hosts (fungi, plants, protists and insects); viruses with and without capsids; and viruses with different numbers of genomic segments. As long as the RdRp phylogeny is adopted as the scaffold for evolutionary reconstruction [6], the mosaic of these other key characteristics of viruses implies complex processes of multiple loss of ancestral genes (in particular, those for capsid proteins), gene exchange between distant viruses and transfer of viruses between distant hosts (such as fungi, plants, animals and protists, but possibly also between bacteria and fungi, even if only via endosymbiosis) [9].

**Figure 1:**
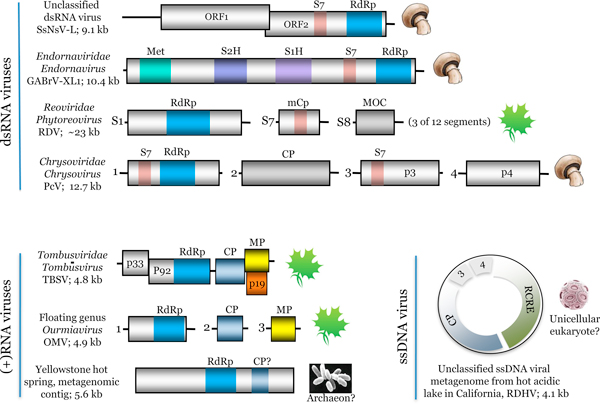
**Mosaic genomes of RNA viruses**. A diagram of the genome architectures for a subset of double-strand RNA (dsRNA), positive-strand RNA ((+)RNA)) and a single-strand DNA (ssDNA) virus discussed in the text. The linear genomes of RNA viruses are shown approximately to scale; the circular ssDNA genome is on a different scale. Black lines correspond to noncoding regions, whereas rectangles designate open reading frames (ORFs), with known protein domains color-coded (RdRp, RNA-dependent RNA polymerase; S7, an RNA-binding domain; Met, methyltransferase/capping enzyme; S1H and S2H, superfamily 1 and 2 helicases, respectively; mCp, minor core protein; MOC, major outer capsid protein; CP, capsid protein; MP, movement protein; p19, suppressor of antiviral RNA interference response; RCRE, rolling-circle replication endonuclease). Proteins encoded in different coding frames are shown with an offset. For the Phytoreovirus, only the three segments relevant for the discussion are shown. Icons on the right show the virus hosts. Virus names are as follows: SsNs-L, *Sclerotinia sclerotiorum nonsegmented virus L*; GABrV-XL1, *Gremmeniella abietina type B RNA virus XL1*; PcV, *Penicillium chrysogenum **v**irus; *RDV, *Rice dwarf virus*; TBSV, *Tomato bushy dwarf virus*; OMV, *Ourmia melon virus*; RDHV, *RNA-DNA hybrid virus*.

Certainly, the phylogeny of the RdRps only reflects the evolutionary history of this gene that is congruent with the histories of other genes only in some limited, tight groups of viruses, but by no account the evolutionary history of viruses themselves. A comprehensive history should integrate the histories of individual genes and can only adequately be conceptualized as a network of multiple evolutionary connections at the level of genes or even parts of genes encoding distinct protein domains. Huiquan Liu and colleagues have made an interesting new contribution to unfolding the network of evolutionary relationships between diverse groups of dsRNA viruses that all have been shown to contain the S7 RNA-binding domain within distinct multidomain proteins (Figure 1). This finding implies that gene transfer between RNA viruses is even more pervasive than previously suspected and further suggests that acquisition of the S7 domain is beneficial for the replication of diverse dsRNA viruses.

Gene exchange that can lead to the emergence of new groups of viruses is not limited to viruses with the same type of genome structure. A recently described group of plant positive-strand RNA viruses, the ourmiaviruses, possess a tripartite genome (Figure 1). One of the segments encodes a predicted RdRp that belongs to a clade that includes the RdRps of fungal narnaviruses and their apparent ancestors, the bacterial leviviruses, whereas the other two segments encode a capsid protein and a movement protein related to the respective proteins of plant viruses, most likely of the family *Tombusviridae *[10]. Thus, in this case, a distinct group of viruses evolved via reassortment of genomic segments derived from extremely diverse viruses, one of which is (at least nominally) a capsid-less dsRNA virus-like replicon whereas the other is a regular positive-strand RNA virus.

Metagenomics, a new major avenue of biological discovery, delivers even more unexpected viral genome arrangements. A recent metagenomic study of the virome of a geothermal lake in California revealed a small circular genome that combines, within one ssDNA segment, genes encoding a capsid protein clearly related to that of plant positive-strand RNA tombusviruses and a rolling circle replication initiation endonuclease homologous to those of ssDNA circoviruses and nanoviruses (Figure 1) [11]. This putative virus - in metagenomics the genome sequence comes before the organism or virus is characterized - seems to be the first ever discovered hybrid between an RNA and a DNA virus (hence appropriately named *RNA-DNA Hybrid Virus*, or RDHV). In this particular case, the genome is in the DNA form and the replication machinery derives from a DNA virus whereas the capsid is of RNA viral origin. It remains to be seen what other surprising chimeras pop up when diverse habitats are explored deeply and systematically.

Beyond discovering strange chimeras, metagenomics has the potential to change the existing fundamental ideas on the evolution of RNA viruses. Almost all extremely diverse RNA viruses infect various eukaryotes. The only two known families of bacterial RNA viruses, *Leviviridae *and *Cystoviridae*, might not even have a common origin with the majority of eukaryotic RNA viruses [6], the evolutionary relationship between *Leviviridae *and *Narnaviridae *notwithstanding. So where does the bulk of RNA viruses come from? A hypothesis has been proposed that the RdRp was derived from the reverse transcriptase of a bacterial retro-transcribing element and the other genes from different bacteriophage and bacterial sources [6]. However, metagenomics has recently offered a viable alternative: positive-strand RNA viruses might have pre-existed in archaeal ancestors of eukaryotes. The first putative archaeal RNA virus has been isolated from near-boiling, archaea-dominated geothermal springs at Yellowstone Park [12]. This novel virus does not belong to any known viral group but encodes a predicted RdRp and capsid protein (Figure 1) that might be ancestral to the respective proteins of eukaryotic positive-strand viruses. Again, in metagenomics, the genome comes before characterization of the virus and its host range, but if the archaeal host is confirmed, the impact of these findings on our understanding of RNA virus evolution will be dramatic.

We are probably only starting to scratch the surface of the virosphere. An unbiased sampling of the viral habitats (and these span the entire biosphere), which with modern technologies is becoming realistic, is expected to result in a major expansion of the networks of virus evolution.
